# Safety and efficacy of the pipeline vantage flow diverter for the treatment of saccular aneurysms in the posterior cerebral circulation

**DOI:** 10.1177/19714009251373070

**Published:** 2025-08-27

**Authors:** Omar Abu-Fares, Antonis Adamou, Peter Raab, Heinrich Lanfermann, Alexander Sirakov, Marie Middendorff, Harold F Hounchonou, Joachim K Krauss, Shadi Al-Afif

**Affiliations:** 1 Institute of Diagnostic and Interventional Neuroradiology, 9177 Hannover Medical School, Germany; 2 Radiology Department, UH St Ivan Rilski, Bulgaria; 3 Department of Neurosurgery, 9177 Hannover Medical School, Germany

**Keywords:** Pipeline vantage flow diverter, posterior circulation aneurysms

## Abstract

**Introduction:**

Posterior circulation aneurysms are particularly challenging to treat due to their anatomical complexity and the high perforator density within this region. The pipeline vantage flow diverter (PVFD) has shown promising results in treating anterior circulation aneurysms. However, its efficacy and safety in treating posterior circulation saccular aneurysms are not well investigated.

**Methods:**

Single-center study reviewed patients with posterior circulation aneurysms treated with the PVFD between September 2021 and March 2024. Patients and aneurysm characteristics, clinical results, and radiological results were documented.

**Results:**

22 patients harboring 24 aneurysms were identified. All aneurysms had a saccular morphology. Complications included ischemic events in two patients (8.3%), one leading to mRS deterioration from 0 to 3. One cerebral hemorrhage leading to mRS shift from 0 to 1 was also documented. At the latest imaging follow-up available (median 14.3 months), complete aneurysm occlusion (Class I, Raymond-Roy occlusion classification (RROC) was achieved in 50% of the cases, residual neck (Class II) in 41.7%, and residual aneurysm (Class III) in 8.3%. Basilar bifurcation aneurysms had lower complete occlusion rates (RROC I: 33.3%) and higher complication rates (16.7% with mRS shift) compared to other locations. In-stent stenosis was rare (4.5%). A limitation of the study is the retrospective, single-center study design.

**Conclusion:**

The PVFD demonstrates high occlusion rates and a favorable safety profile in the treatment of saccular aneurysms in the posterior circulation. However, treatment of basilar bifurcation aneurysms with the PVFD remains challenging due to the complex anatomy and high-flow dynamics in this location.

## Introduction

Posterior circulation aneurysms refer to aneurysms arising from the vertebrobasilar system, including the basilar artery, vertebral arteries, and posterior cerebral arteries.^1,2^ They account for approximately 10%–15% of all intracranial aneurysms and are located near the brainstem making surgical and endovascular treatment options challenging.^3,4^ Aneurysm location within the posterior circulation was identified as a predictor of poor outcome for electively treated patients, involving both surgical and endovascular techniques due to their proximity to cranial nerves, and perforating arteries.^1,2,5,6^ Moreover, the morphology of these aneurysms, including broad necks and complex branching patterns, often limits the use of standard endovascular treatment techniques.^7,8^

While endovascular therapy is typically preferred over surgical clipping for most posterior circulation aneurysms, traditional techniques such as primary coil embolization as well as stent- or balloon-assisted coiling often prove inadequate for effectively treating some of these aneurysms.^9^ Since flow diversion with the pipeline embolization device has been developed for the treatment of complex aneurysms, some reports have emerged describing the use of flow diverters (FDs) in the treatment of posterior circulation aneurysms with promising results.^10,11^

The fourth generation pipeline vantage flow diverter (PVFD) is safe and effective for the treatment of saccular intracranial aneurysms, particularly in the anterior circulation with complete aneurysm occlusion rate of 74.3% at 6 months follow-up and thromboembolic complications of 6.1%.^12^ To our knowledge, this study is the first to exclusively investigate the use of the PVFD for the treatment of saccular non-ruptured aneurysms of the posterior circulation.

## Methods

The study was conducted in compliance with the regulations of the local ethical committee at our institution. Written informed consent was obtained from all patients included in the study. We retrospectively reviewed our database to identify all patients with intracranial saccular aneurysms in the posterior circulation treated with the PVFD between September 2021 and March 2024. Inclusion criteria for this series were all saccular aneurysms treated primarily with the PVFD only or aneurysm recurrence following prior coiling. Only patients with unruptured aneurysms or past the acute stage (>90 days) of subarachnoid hemorrhage (SAH) were included. Fusiform, dissecting, and blister-like aneurysms were excluded. Aneurysms treated with other parent vessel implants such as other FDs or stents were also excluded. Descriptive statistics and frequency distribution were evaluated.

## Device description

The fundamental component of the FD stent design is a braided mesh. The mesh’s porosity, combined with the pressure gradient between the main vessel and smaller adjacent vascular branches, helps maintain blood flow and patency in these branches, even when covered by the device. This is particularly important in vessels with a high density of perforators, which are commonly found in the posterior circulation.

An effective FD should have ideal pore density and firm wall apposition to encourage blood flow stagnation within the aneurysm.^13^ A thinner implant profile aids in promoting endothelial coverage of the device, while reduced thrombogenic features of the implant minimize the risk of ischemic complications.

The PVFD with Shield Technology is a novel (fourth generation) FD constructed from either 48 cobalt-chromium wires (for 2.50–3.50 mm device diameter) or 64 wires (for 3.50–6.00 mm device diameter). The 64-wire version includes additional 16 solid cobalt-chromium wires to enhance radial force. Notably, these wires are thinner than those of the third-generation Pipeline Shield, resulting in a reduced overall thickness of the device. Moreover, the current technical modification of the PVFD might bring improvements, which could make device deployment easier and more efficient. Due to the redesigned expanded polytetrafluoroethylene (ePTFE) sleeves, the distal portion of the device opens reliably with simple unsheathing—regardless of its size—without the need for techniques like re-sheathing or “open-and-drag.” Therefore, shifting from a pushing technique to unsheathing may lead to better wall apposition, potentially reducing the need for additional steps after deployment, such as “wagging,” balloon/stent angioplasty, or J-wire opening maneuvers. The phosphorylcholine surface modification of the device provides similar clinical outcomes regarding aneurysm occlusion compared to prior pipeline FD generations while enhancing the safety profile by lowering the thrombogenic features of the implant and potentially promoting early neointimal coverage.^14–16^

## Endovascular procedure and dual antiplatelet therapy (DAPT)

All procedures were carried out under general anesthesia via a femoral or radial arterial access using a Canon Alphenix Sky + biplane digital subtraction angiography (DSA) system (Canon Medical Systems, Neuss, Germany). Patients were heparinized with a bolus of 5000 IU unfractionated heparin (IVCO Healthcare LLC, Mongolia). Typically, a 6F guiding catheter was placed in the proximal vertebral artery. A distal access catheter (DAC) was then advanced in to the V4 segment of the vertebral artery proximal to the aneurysm. Next, a 0.027″ or 0.021″ microcatheter was advanced into the parent vessel distal to the aneurysm. Then, a PVFD was deployed in the landing zone (at least 3 mm distal to the aneurysm neck) of the parent vessel covering the aneurysm neck. The endovascular procedure ended with DSA documentation in the working and standard projections as well as flat panel volume CT angiography.

The DAPT protocol consisted of a loading dose of 100 mg acetylsalicylic acid (ASA) and 600 mg clopidogrel given the night before the procedure, and a repeat loading dose of 100 mg ASA and 300 mg clopidogrel at the morning of the procedure. Starting the day after the procedure, patients received a daily maintenance regimen of 100 mg ASA and 75 mg clopidogrel. The medication was continued for 3 months and followed by monotherapy of 100 mg ASA daily.

## Results

### Characteristics of patients, aneurysms and PVFD

Between September 2021 and March 2024, 22 patients harboring 24 aneurysms were treated. The median age of the patients was 57 years (range: 37–79 years), and 50% (*n* = 11) of patients were female. Patient data and comorbid conditions are summarized in Table 1.

**Table 1.** Summary of patient data, aneurysm, and flow diverter characteristics.

Characteristics	Frequency
Patient data	*n* = 22
Age, median (range)	57 (37–79)
Female, *n* (%)	11 (50%)
Baseline mRS, median (range)	0 (0–4)
BMI, median (range)	26.67 (17.9–26.6)
Arterial hypertension, *n* (%)	15 (68.2%)
Smoking history, *n* (%)	10 (45.5%)
Diabetes mellitus, *n* (%)	1 (4.5%)
Aneurysms treated, *n*	24
Previously coiled, *n* (%)	4 (18.2%)
Aneurysm characteristics	Median in mm (range)
Aneurysm diameter	5.75 (1.5–13)
Aneurysm height	5 (2–12)
Aneurysm neck diameter	4.25 (1.5–11)
Artery and PVFD characteristics	Median in mm (range)
Maximum vessel width	3.5 (2.5–5.5)
Minimum vessel width	2.5 (1.5–4)
PVFD length	16 (12–40)
PVFD width	3.5 (2.75–5.5)
Aneurysm location	*n* (%)
Vertebral artery(V4 segment)	3 (12.5%)
Posterior inferior cerebellar artery (PICA)	4 (16.6%)
Superior cerebellar artery	7 (29.2%)
Basilar trunk	3 (12.5%)
Basilar bifurcation	6 (25%)
Posterior cerebral artery	1 (4.2%)

*n*: number, PICA: posterior inferior cerebellar artery, PVFD: pipeline vantage flow diverter.

Of the 24 aneurysms, 4 (16.6%) had been previously coiled for the treatment of aneurysmal SAH. All aneurysms had a saccular morphology and were located in the posterior circulation. Summary of aneurysm characteristics and the PVFD used are shown in Table 1.

### Clinical outcomes and complications

**Figure 1.**
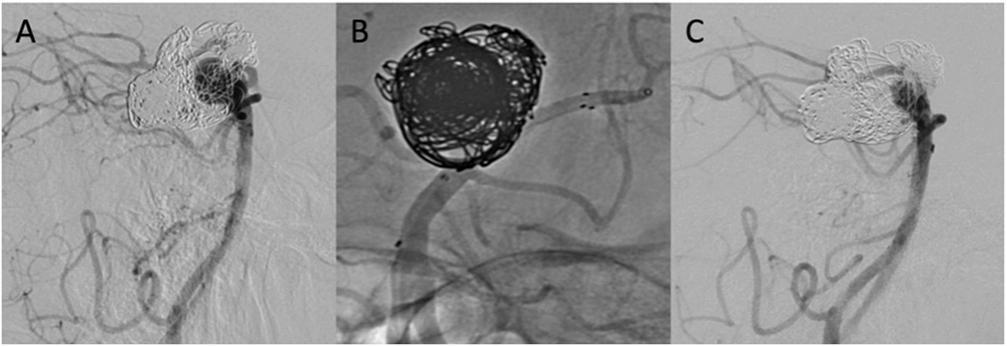
Pipeline vantage flow diverter (PVFD) implantation for the treatment of a re-vascularized saccular aneurysm at the basilar bifurcation after previous coil embolization in a 53-year-old male patient. Digital subtraction angiography (DSA) lateral view shows a saccular aneurysm of the basilar tip with recanalization of prior stent-assisted coiling (A). A microcatheter is advanced through the Neuroform Atlas stent before PVFD deployment (B). At 15-month follow-up, the DSA lateral view shows reduction of the aneurysm size with persistent aneurysm opacification (C).

Clinical follow-up was available for all patients at a median of 21 months (range: 8–32 months). Before the intervention, 20 (90.9%) patients had a mRS of 0, 1 (4.5%) patient a mRS of 4, and 1 (4.5%) patient a mRS score of 3. Immediately after the intervention, one patient suffered a compartment syndrome of the forearm after radial puncture, which required surgical intervention. At the latest clinical follow-up 2 years after the intervention, mild deficit of the forearm was documented. Two other patients, who were treated with the PVFD for the treatment of basilar bifurcation aneurysms, suffered ischemic events (Figure 1), resulting in persistent hemiparesis in one case and reversible symptoms of diplopia and nausea in the other case. At the latest clinical follow-up available, 17 (77.3%) patients had a mRS of 0 and 3 (13.6%) patients experienced mRS deterioration. Patients with initial mRS scores of 3 and 4 did not experience a mRS shift throughout the follow-up period. No mortality occurred. All complications are summarized in Table 2.

**Table 2.** Summary of complications following PVFD implantation.

Sex, age	Description	mRS shift (baseline *→* follow-up)	Aneurysm location	PVFD size (width × length in mm)
Female, 49	Asymptomatic in-stent stenosis; spontaneous regression at 9-month follow-up	No change	SCA	4 × 14
Female, 62	Brainstem infarction 6 days following the intervention	0–3	Basilar bifurcation	3.5 × 25
Female, 58	Peri-interventional thromboembolic complication with reversible symptoms (nausea and diplopia)	No change	Basilar bifurcation	4.5 × 20
Female, 57	Spontaneous cerebral hemorrhage 1 month after the intervention requiring surgical evacuation	0–1	SCA	3 × 20
Male, 65	Compartment syndrome of the forearm following radial puncture	0–2	SCA	5 × 16

PVFD: pipeline vantage flow diverter; SCA: superior cerebellar artery; mRS: modified Rankin scale.

### Radiological outcomes

Radiological follow-up was available for all patients at a median of 14.3 months (range: 2.3–26.6 months). The aneurysm occlusion rate was classified according to the Raymond-Roy occlusion classification (RROC). Class I (complete occlusion) was observed in 12 (50%) patients, Class II (neck remnant) in 10 (41.7%) patients, and Class III (aneurysm remnant) in 2 (8.3%) patients. Aneurysm occlusion rates according to the different anatomical locations within the posterior circulation are summarized in Table 3.

**Table 3.** Radiological outcome.

Grade of aneurysm occlusion (Raymond-Roy classification) and aneurysm location	Median radiological follow-up in months (range)	*n* = 24
Class I (complete occlusion), *n* (%)	10 (3–26)	12 (50%)
PICA, 3 (75%)		
Basilar trunk, 2 (66.6%)		
SCA, 5 (71.4%)		
V4, 2 (66.6%)		
Class II (neck remnant), *n* (%)	13.5 (3–20)	10 (41.7%)
PICA, 1 (25%)		
Basilar trunk, 1 (33.3%)		
SCA, 2 (28.6%)		
V4, 1 (33.3%)		
Basilar bifurcation, 4 (66.7%)		
Posterior cerebral artery, 1 (100%)		
Class III (aneurysm remnant), *n* (%)	9.5 (3–16)	2 (8.3%)
Basilar bifurcation, 2 (33.3%)		

PICA: posterior inferior cerebellar artery, SCA: superior cerebellar artery.

**Figure 2.**
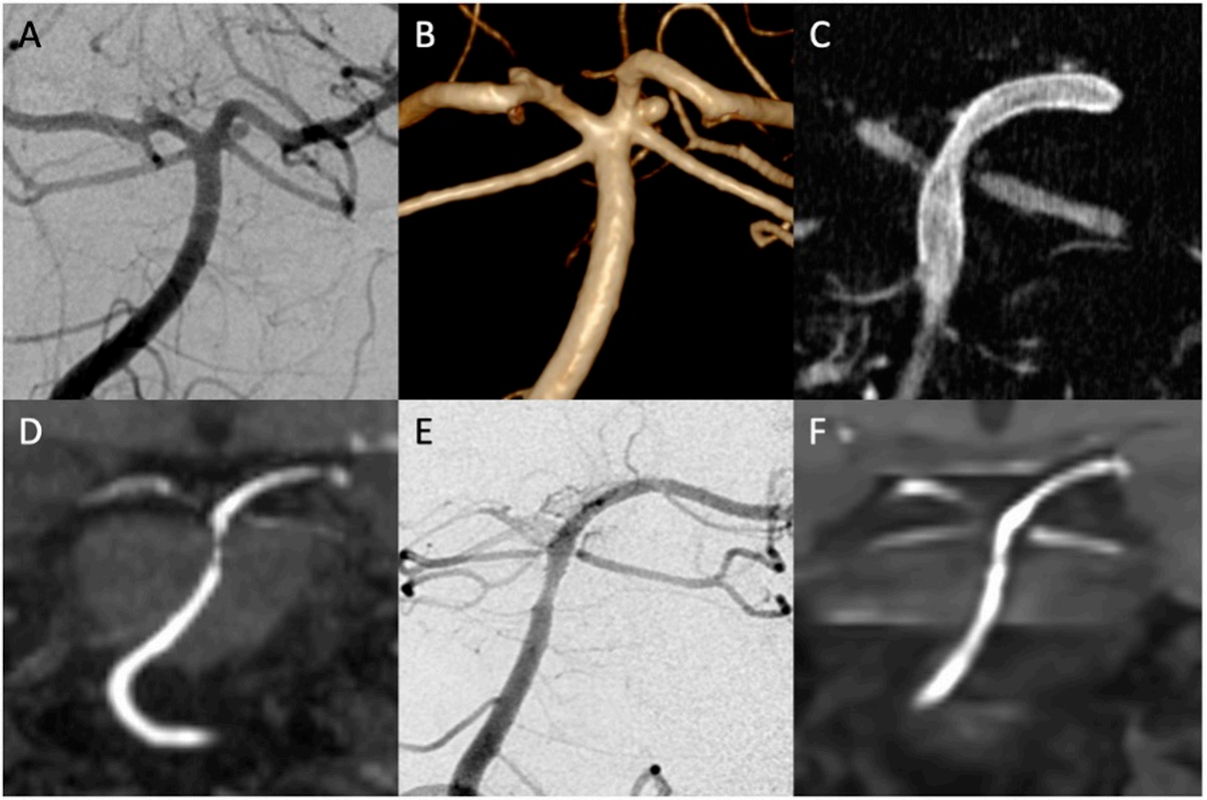
Pipeline vantage flow diverter (PVFD) implantation for the treatment of an unruptured saccular superior cerebellar artery (SCA) aneurysm. Digital subtraction angiography (DSA) and 3D reconstruction of a rotational angiography anterior view (A and B) show a saccular aneurysm located at the origin of the left SCA in a 49-year-old female. After positioning of a Phenom 21 microcatheter in the left posterior cerebral artery, a 3 × 12 mm PVFD was implanted as shown in the flat panel computer tomography(C). On the control MRI 3 months after the treatment, the aneurysm was completely occluded (D). Moderate thickening of the vessel wall with moderate in-stent stenosis was noted at the middle portion of the PVFD. DSA and MRI follow-up 6 months after PVFD implantation show residual slight in-stent stenosis (E and F).

**Figure 3.**
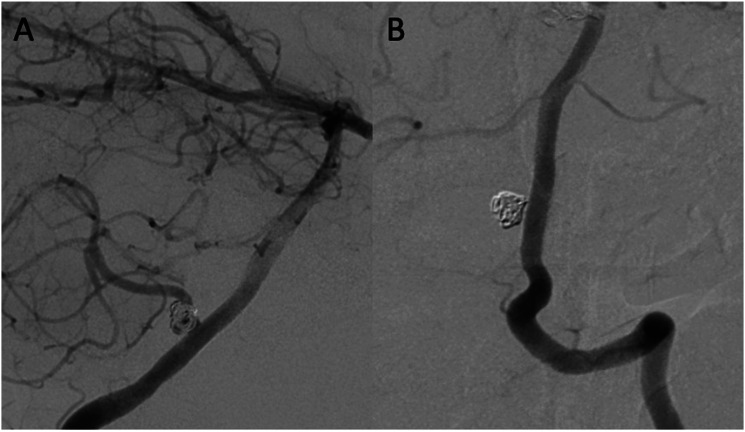
Pipeline vantage flow diverter (PVFD) implantation for the treatment of a re-vascularized aneurysm located at the origin of the posterior inferior cerebellar artery (PICA) in a 44-year-old female. Digital subtraction angiography (DSA) lateral view shows a saccular aneurysm at the origin of the left PICA with recanalization after coiling for aneurysmal subarachnoid hemorrhage (A). At follow-up 18 months after PVFD implantation, DSA shows occlusion of the left PICA without clinical sequelae (B).

In-stent stenosis (ISS) was observed in one case (4.5%) at the 3-month MRI follow-up after PVFD implantation for the treatment of a superior cerebellar artery (SCA) aneurysm. DAPT was extended, and another DSA performed 9 months after the intervention showed improvement of the ISS (Figure 2). Moreover, 3 (75%) patients with aneurysms located at posterior inferior cerebellar artery (PICA) origin treated with PVFD implantation in the V4 segment of the vertebral artery developed occlusion of the PICA, which was detected on routine follow-up imaging. Since all PICA occlusions occurred without clinical sequelae, DAPT was discontinued as scheduled (Figure 3).

## Discussion

The present study reports high occlusion rates for posterior circulation saccular aneurysms treated with PVFD. This rate is comparable to the occlusion rates observed in anterior circulation aneurysms as reported in previous studies.^12,17,18^ The existing body of literature on PVFDs predominantly focuses on aneurysms located within the anterior cerebral circulation; pooled data from 5 studies (373 patients or 418 aneurysms) demonstrated that the majority of treated aneurysms are situated in the anterior circulation, particularly along the ICA.^19^ This trend likely reflects the long-standing belief that flow diversion is best suited for sidewall aneurysms of the internal carotid artery (ICA). In contrast, using FDs in the posterior circulation is generally viewed as more complex, largely due to the smaller vessel diameters and the high density of perforating arteries in this region—factors that increase the risk of delayed ischemic complications following treatment.

This report investigates the use of the PVFD for treatment of saccular aneurysms in the posterior circulation. Most previous studies investigated the PVFD in groups of patients with heterogeneous aneurysm locations and types, including only a small percentage of aneurysms in the posterior circulation, or involving various types of aneurysms (saccular, fusiform, and dissecting).^20^ Booth et al. reported a smaller proportion of posterior circulation aneurysms compared to anterior circulation aneurysms without specifying the exact number and including both saccular and non-saccular aneurysms without providing information about the aneurysm location in the posterior circulation. The study showed an overall occlusion rate of >90% for all aneurysms, but posterior circulation aneurysms were associated with higher complication rates.^18^ In the study from De Villiers et al., 11/115 aneurysms (9.6%) were located in the posterior circulation, without including details about aneurysm type. The overall aneurysm occlusion rate at 6 months was 81.7%, yet the aneurysm occlusion rates for the various locations within the posterior circulation were not separately reported.^17^ Another study from Vollherbst et al. reported on 7/70 (10%) predominantly saccular aneurysms located in the posterior circulation, which were treated with PVFD. The study reported a complete occlusion rate of 77.9% after 7 months follow-up for all aneurysms combined, nonetheless not providing aneurysm occlusion rates for posterior circulation aneurysms separately.^21^ Goertz et al. reported 8/32 aneurysms (31%) in their series located in the posterior circulation, of which seven were fusiform and one was dissecting. The complete occlusion rate for posterior circulation aneurysms was 27% at 6 months, with an overall occlusion rate of 47%.^22^ Across the studies, the number of posterior circulation aneurysms ranged from 7 to 11 cases, accounting for 10%–31% of the total aneurysms treated. In contrast, our study included a larger cohort of patients with only one type of aneurysm (saccular) in the posterior circulation, which makes statements regarding safety and efficacy for this type of aneurysm more reliable.

Our data reveals a lower aneurysm occlusion rate for basilar bifurcation aneurysms treated with the PVFD in comparison to other posterior circulation locations, which might be due to challenges uniquely associated with bifurcation aneurysms. Previous studies have suggested that the complex branching anatomy and high-flow dynamics at the basilar bifurcation pose significant challenges for optimal device apposition and effective sustained flow diversion.^23^ This emphasizes the need for procedural techniques or design adaptations of the device tailored to this particular anatomy. It is also possible that the follow-up period available in our study was not long enough, as these aneurysms may require longer time to completely occlude after flow diversion.

In our study, intradural vertebral artery aneurysms (V4 segment) treated with the PVFD demonstrated favorable outcomes with high occlusion rates and no complications. This may be attributed to the low density of perforators in this region. Aneurysms at the PICA origin also had good outcomes with no symptomatic complications, despite secondary occlusion of the PICA in 75% of cases. The absence of complications may be explained by the development of collateral blood supply from the anterior inferior cerebellar artery (AICA). Basilar trunk sidewall aneurysms also had good occlusion rates and no complications after flow diversion. Similarly, saccular aneurysms of the SCA region had high occlusion rates after PVFD implantation, with two non PVFD-related complications recorded. The moderate complication rate aligns with reported outcomes in previous studies, where use of antiplatelet therapy has been emphasized.^12,22^

PVFD had an acceptable complication profile in our cohort, with lower rates of ischemic and hemorrhagic events as compared to other FDs.^10^ Sciacca et al. had reported substantial complications, particularly in ruptured posterior circulation aneurysms where mortality reached up to 20%.^24^ The different types of aneurysms treated (non-acutely ruptured and saccular aneurysms) in comparison to the above-mentioned studies might explain the lower complication rate in our cohort.

A notable finding in our study is the relatively low incidence of ISS after PVFD implantation in the posterior circulation. This result stands in contrast to higher ISS rates observed in anterior circulation cases in previous studies.^12,17^ It is possible that neointimal growth and the anatomical and hemodynamic differences between posterior and anterior circulation vessels account for this disparity.

Our findings further underscore the importance of DAPT in mitigating thromboembolic risk during and after FD treatment, even with the new PVFD’s modified surface. Consistent with findings from Sciacca et al., and de Villiers et al., our study indicates that DAPT is instrumental in achieving low thromboembolic rates after PVFD implantation in the posterior circulation.^17,24^ Compared to the p64 Flow Modulation Device examined by Hellstern and colleagues, which was accompanied by a relatively high complication rate in saccular posterior circulation aneurysms,^10^ the PVFD demonstrated lower complication rate in our study. The PVFD’s thinner implant profile and phosphorylcholine coating may be contributing factors, as they reduce thrombogenic potential. These findings suggest that the PVFD might offer a safer alternative for posterior circulation aneurysms, especially in perforator-rich regions.

The limitations are the study’s single-center design, a relatively small sample size as well as a wide range of follow-up available. Future multicenter, randomized trials are needed to confirm the PVFD’s efficacy and safety across varied patient populations and aneurysm types. Additionally, more research into tailored DAPT protocols for posterior circulation aneurysms could optimize safety, balancing thromboembolic protection with hemorrhagic risk reduction.

## Conclusion

The PVFD demonstrates high occlusion rates with a favorable complication profile, supporting its use as a treatment option for non-ruptured, saccular aneurysms in the posterior circulation. The lower occlusion rates of aneurysms located at the basilar bifurcation highlights the importance of considering anatomical peculiarities.

## Appendix

### Abbreviations

ASA: Acetylsalicylic acid
AICA: Anterior inferior cerebellar artery
CT: Computer tomography
DAPT: Dual antiplatelet therapy
DAC: Distal access catheter
DSA: Digital subtraction angiography
FD: Flow diverter
ICA: Internal carotid artery
ISS: In-stent stenosis
MRI: Magnetic resonance imaging
mRS: Modified Rankin Scale
NIH: Neointimal hyperplasia
PICA: Posterior inferior cerebellar artery
PVFD: Pipeline vantage flow diverter
RROC: Raymond-Roy occlusion classification
SAH: Subarachnoid hemorrhage
SCA: Superior cerebellar artery.
